# Standardized Environmental Enrichment Supports Enhanced Brain Plasticity in Healthy Rats and Prevents Cognitive Impairment in Epileptic Rats

**DOI:** 10.1371/journal.pone.0053888

**Published:** 2013-01-14

**Authors:** Raafat P. Fares, Amor Belmeguenai, Pascal E. Sanchez, Hayet Y. Kouchi, Jacques Bodennec, Anne Morales, Béatrice Georges, Chantal Bonnet, Sandrine Bouvard, Robert S. Sloviter, Laurent Bezin

**Affiliations:** 1 Université de Lyon, Lyon, France; 2 Université Lyon 1, Villeurbanne, France; 3 Inserm, Institut National de la Santé et de la Recherche Médicale, U1028, Lyon Neuroscience Research Center, Tiger Team, Lyon, France; 4 CNRS, Centre National de la Recherche Scientifique, UMR5292, Lyon Neuroscience Research Center, Tiger Team, Lyon, France; 5 IDÉE, Institut Des ÉpilepsiEs, Lyon, France; 6 IRBA, Institut de Recherche Biomédicale des Armées, Brétigny-sur-Orge, France; 7 Department of Neurobiology, Pharmacology and Toxicology, Morehouse School of Medicine, Atlanta, Georgia, United States of America; University of North Dakota, United States of America

## Abstract

Environmental enrichment of laboratory animals influences brain plasticity, stimulates neurogenesis, increases neurotrophic factor expression, and protects against the effects of brain insult. However, these positive effects are not constantly observed, probably because standardized procedures of environmental enrichment are lacking. Therefore, we engineered an enriched cage (the Marlau™ cage), which offers: (1) minimally stressful social interactions; (2) increased voluntary exercise; (3) multiple entertaining activities; (4) cognitive stimulation (maze exploration), and (5) novelty (maze configuration changed three times a week). The maze, which separates food pellet and water bottle compartments, guarantees cognitive stimulation for all animals. Compared to rats raised in groups in conventional cages, rats housed in Marlau™ cages exhibited increased cortical thickness, hippocampal neurogenesis and hippocampal levels of transcripts encoding various genes involved in tissue plasticity and remodeling. In addition, rats housed in Marlau™ cages exhibited better performances in learning and memory, decreased anxiety-associated behaviors, and better recovery of basal plasma corticosterone level after acute restraint stress. Marlau™ cages also insure inter-experiment reproducibility in spatial learning and brain gene expression assays. Finally, housing rats in Marlau™ cages after severe *status epilepticus* at weaning prevents the cognitive impairment observed in rats subjected to the same insult and then housed in conventional cages. By providing a standardized enriched environment for rodents during housing, the Marlau™ cage should facilitate the uniformity of environmental enrichment across laboratories.

## Introduction

Housing animals in conventional cages models a sedentary lifestyle with poor cognitive stimulation, while raising animals in enriched cages is a better model of an active lifestyle with a greater level of cognitive and sensorimotor stimulation [1], [2], [3], [4]. In experimental models of human diseases that involve live animals, the interaction between the quality of the living environment and the genetic risk factors is critical in the etiology and progression of diseases [2], [5], [6], [7] and may affect the response to potential therapeutics. Thus, there is an elevated risk that neglecting this interaction in preclinical studies undermines the success of clinical trials.

Today, preclinical studies in joint research programs may require multidisciplinary approaches involving laboratories and research groups sometimes located at long distances from each other. Thus, standardization of housing procedures is a fundamental requirement for both conventional and enriched paradigms, aimed at producing, at multiple sites, animals exhibiting a similar basal state for all measured variables. Standardization of conventional housing conditions can be easily achieved through diverse equipments available on the market, in accordance to the guidelines of various international regulatory agencies. By contrast, despite important guidelines aimed at harmonizing and standardizing environmental enrichment for rodents [8], [9], no cage is currently available to fulfill this purpose. Therefore, we developed the Marlau™ cage to standardize the procedures of environmental enrichment for rodents, meeting not only the principles of animal welfare research, aimed at developing rodent-specific behavior, but also the principles of neuroscience research whose objectives are to increase social and sensory stimulations in order to evoke brain and cognitive reserve, to support functional rehabilitation after brain insults and to produce better resistance in drug addiction [2], [3], [10], [11], [12].

The Marlau™ cage includes a series of mazes, the configuration of which is changed regularly, thus supporting complexity and novelty. In addition, we ensured that all animals were exposed to maze training by placing mazes between food pellet and water bottles compartments. All the description of the Marlau™ cage has been previously described in great details [13]. Here, we provide evidence that housing in Marlau™ cages meets the criteria of enrichment-induced beneficial effects on brain function in physiological and pathological conditions.

## Results

### General Behavioral Observation

The most fundamental feature of the Marlau™ cage is the maze separating food pellets and water bottles compartments [13]. We needed to ascertain that change in maze configuration, that occurred three times a week, was a thorough source of curiosity. Rats were observed during the one-hour period after each maze change. They all moved with a high level of activity throughout the maze after each configuration change until they found the exit, and then returned back to it for ∼30 min to explore all alleys with both horizontal and vertical movements. After that time, rats ate food pellets within the maze, after carrying them from the G1 ground compartment. The day when both bedding material and maze configuration were changed, rats climbed first to the maze until they found the exit, and then explored intensively the ground floor for ∼20 min. Most rats started to visit the maze more in details once general activity decreased in the ground floor. During ground exploration, social activity, including play-fighting behavior, was enhanced.

### Body Weight, Food Intake and Total Lipids

**Figure 1:**
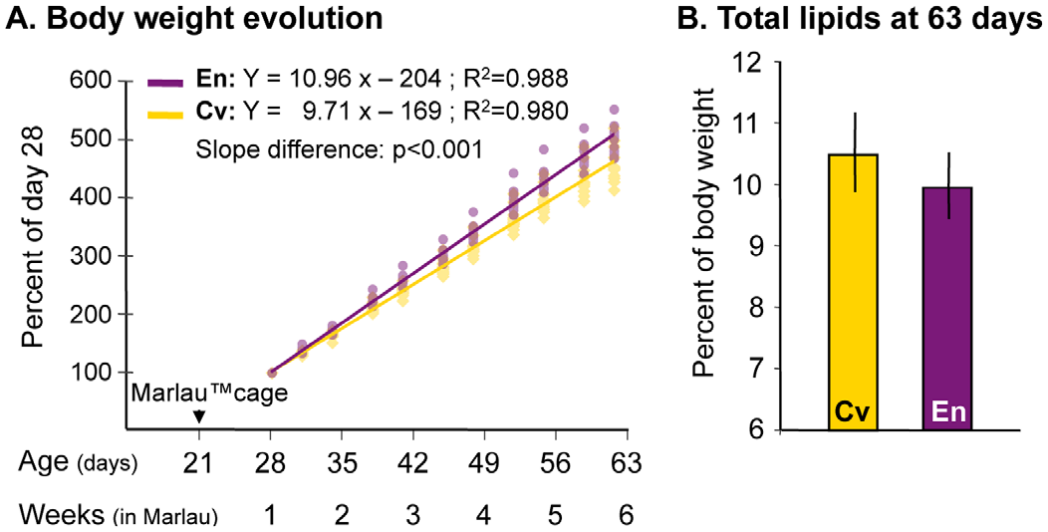
Rat weight, but not total body fat, is increased after housing in the Marlau™ cage. A. After 4 weeks in the Marlau™ cage, “enriched” rats had an increase in body weight that was significantly greater than that of rats raised in conventional conditions (n = 12 in each group). **B.** Total body fat percentage measured at termination time was not altered by housing conditions (n = 8 in each group). All results are expressed as the mean ± SEM. Abbreviations: Cv, rats in conventional cages; En, rats in Marlau™ cages.

Because enriched housing in Marlau™ cages started at weaning in our paradigm, we first verified whether housing conditions (enriched compared to conventional) altered body growth. We show that body weight gain in rats raised in Marlau™ cage (enriched group, En) was greater than that of rats raised in conventional cages (conventional group, Cv): +12.9% (p<0.001, Student’s *t*-test on the slope) from day 28 to day 63 (Fig. 1A). The body weight gain in En rats was associated with greater food intake (+7.4% from weeks 1 to 6 in Marlau™ cages, data not shown), with no modification of total body lipid proportion (Fig. 1B). The body weight gain in En rats was confirmed in the other experiments: +11.5%, +9.8%, +10.8 and +11.2% in experiments 2, 3a, 3b and 4, respectively.

### Restraint Stress Coping

**Figure 2:**
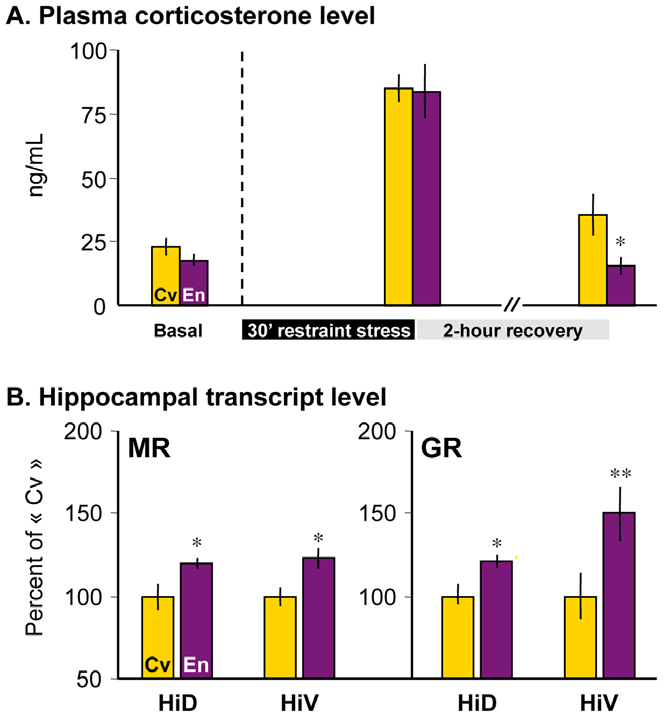
Rats raised in Marlau™ cages display better capacity to cope with restraint stress. A. Plasma corticosterone level was measured 30 min prior to (basal), at the end of a 30 min-restraint stress and after 2-hour recovery period (n = 5 in each group). En vs. Cv: * p<0.05, following two-way repeated measures ANOVA. **B.** Transcript levels for corticosteroid receptors (MR and GR) were measured in the dorsal and ventral hippocampus of naïve rats raised in conventional or Marlau™ cages (n = 7 in each group). Note that increased hippocampal transcript level of GR and MR was associated with a faster recovery of basal CORT level after restraint stress. En vs. Cv: * p<0.05, ** p<0.01, following two-way ANOVA, factor 1: rat group, factor 2: hippocampal subregion. Abbreviations: Cv and En as in Fig. 1.

The basal plasma level of CORT was similar between rats raised in Marlau™ cages and those raised in conventional cages (Fig. 2A). Peak plasma CORT level after 30 min of restraint stress was also similar in rats raised in the two housing conditions, but recovery of basal plasma level of CORT was faster in rats raised in Marlau™ cages than in rats raised in conventional cages, as measured 2 hours after stress cessation (Fig. 2A).

One of the brain areas involved in the recovery of baseline level of plasma CORT after a stressful condition is the hippocampus. CORT binds to mineralocorticoid (MR) and glucocorticoid (GR), and both receptors are present in the hippocampus [14], [15]. MRs have a high affinity for CORT and are thought to regulate the basal activity of the hypothalamic-pituitary-adrenal system. Conversely, GRs have low affinity for CORT, and hippocampal GRs are thought to play an important role in the recovery of baseline plasma levels of CORT after high release of CORT as seen during acute stress [14], [15]. Here, we show that MR and GR transcript levels in the dorsal and ventral hippocampus of “enriched” rats were greater than that of “conventional” rats (table 1; Fig. 2B). Similar results were previously obtained for GR in the dorsal hippocampus [16]. These results indicate that hippocampal upregulation of MR and GR gene expression in rats raised in Marlau™ cages might participate in faster recovery of basal plasma CORT level after exposure to an acute stress.

**Table 1:** Number of cDNA copies (mean ± SEM) after reverse transcription of 1 µg of total RNAs contained in the ventral and dorsal hippocampus dissected from the brains of 9 week-old rats housed in conventional cages (n = 8).

	Dorsal Hippocampus	Ventral Hippocampus	
BDNF	1,109,200±63,320	428,280±18,000	***
Epo	13,640±116	14,320±2,480	NS
EpoR	55,120±4,760	20,720±680	***
GR	7,504,040±382,560	2,303,800±323,080	***
IGF-1	412,240±18,800	227,080±4,840	***
MAP-2	1,157,800±50,960	464,720±81,760	***
MR	2,109,520±163,560	609,960±34,680	***
TrkB.FL	983,080±43,200	426,920±47,520	***
TrkB.T1	399,960±25,120	179,240±2,640	***
TrkB.T2	2,040±764	600±76	NS
VEGF	375,760±30,812	217,320±12,387	***

Differences between dorsal and ventral hippocampus:

*** p<0.001, Student’s *t* test;

NS: not significantly different.

### Cortical Thickness and Hippocampal Surface Area

**Figure 3:**
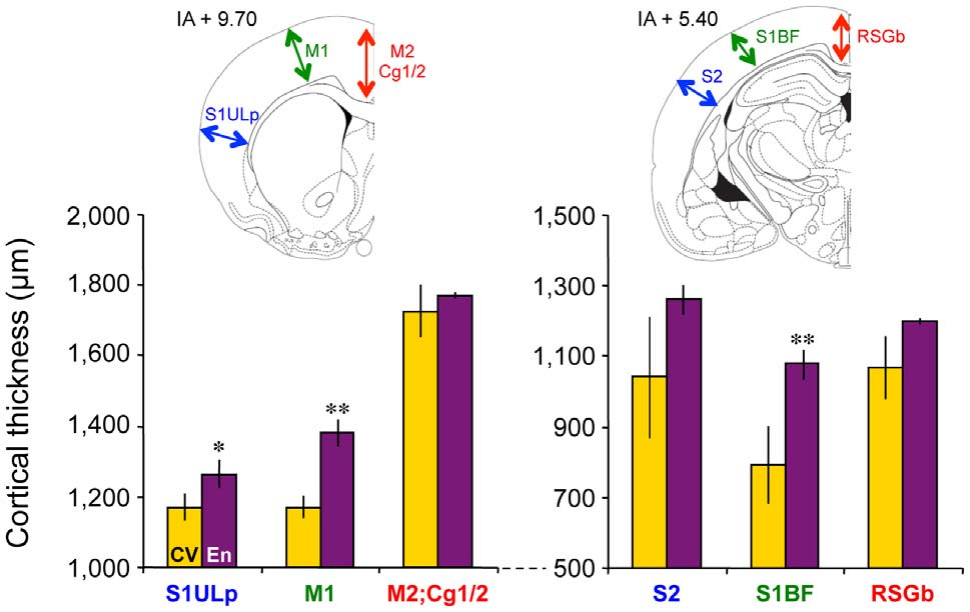
Marlau™ cages meet the criterion of enrichment-induced cortical thickness. Six weeks after housing in Marlau™ cages, averaged cortical thickness measured as shown by colored arrows on Nissl-stained sections was increased compared to conventional conditions, especially in M1 and S1 subregions. En vs. Cv: * p<0.05, ** p<0.01, two-way ANOVA, factor 1: rat group, factor 2: subregion. Each bar represents the mean ± SEM (*n = 4* in each group). Abbreviations: Cv and En as in Fig. 1; Cg1/2, cingular cortex 1 and 2; M1, primary motor cortex; M2, secondary motor cortex; RSGb, retrosplenial granular b cortex; S1BF, primary somatosensory cortex, barrel field; S1ULp, primary somatosensory cortex, upper lip region.

The Marlau™ cage was designed, in part, to enhance voluntary exercise (large surfaces to explore and the presence of running wheels) and motivated exercise (exploratory behavior in the maze to find food at each maze change). Exercise has been shown to increase the thickness of primary motor cortex (M1) and primary somatosensory cortex (S1) [17]. Therefore, we determined whether the thicknesses of M1 and S1 were increased in rats raised in Marlau™ cages. From Nissl-stained coronal brain sections taken at 120 µm intervals, two sections of each brain were selected at coronal planes corresponding to IA +9.70 and +5.40 according to Paxinos and Watson [18]. We measured cortical thickness, as illustrated (Fig. 3) in three different subregions on each section: cingular cortex 1 and 2 and secondary motor cortex (Cg1/2;M2), primary motor cortex (M1) and primary somatosensory cortex, upper lip region (S1ULp) at IA +9.70 mm, and retrosplenial granular b cortex (RSGb), primary somatosensory cortex, barrel field (S1BF) and secondary motor cortex (M2) at IA +5.40 mm. Although there was a tendency for an overall increase in cortical thickness, a statistically significant increase in thickness was found only in the M1 and S1 (S1ULp and S1BF) subregions (Fig. 3).

Surface area measurements of the hippocampus (both sides) were performed at 240 µm intervals from IA +5.86 mm to IA +4.90 mm in the dorsal anterior hippocampus (daHi), and from IA +4.20 mm to IA +3.72 mm in the dorso-ventral hippocampus (dvHi). We did not detect any differences in the surface area of the hippocampus between rats raised in conventional and enriched cages (daHi, Cv: 30.29±2.05 mm^2^, En vs. Cv: 100±1%; dvHi, Cv: 50.07±2.30 mm^2^, En vs. Cv: 88±10%).

### Neurogenesis

**Figure 4:**
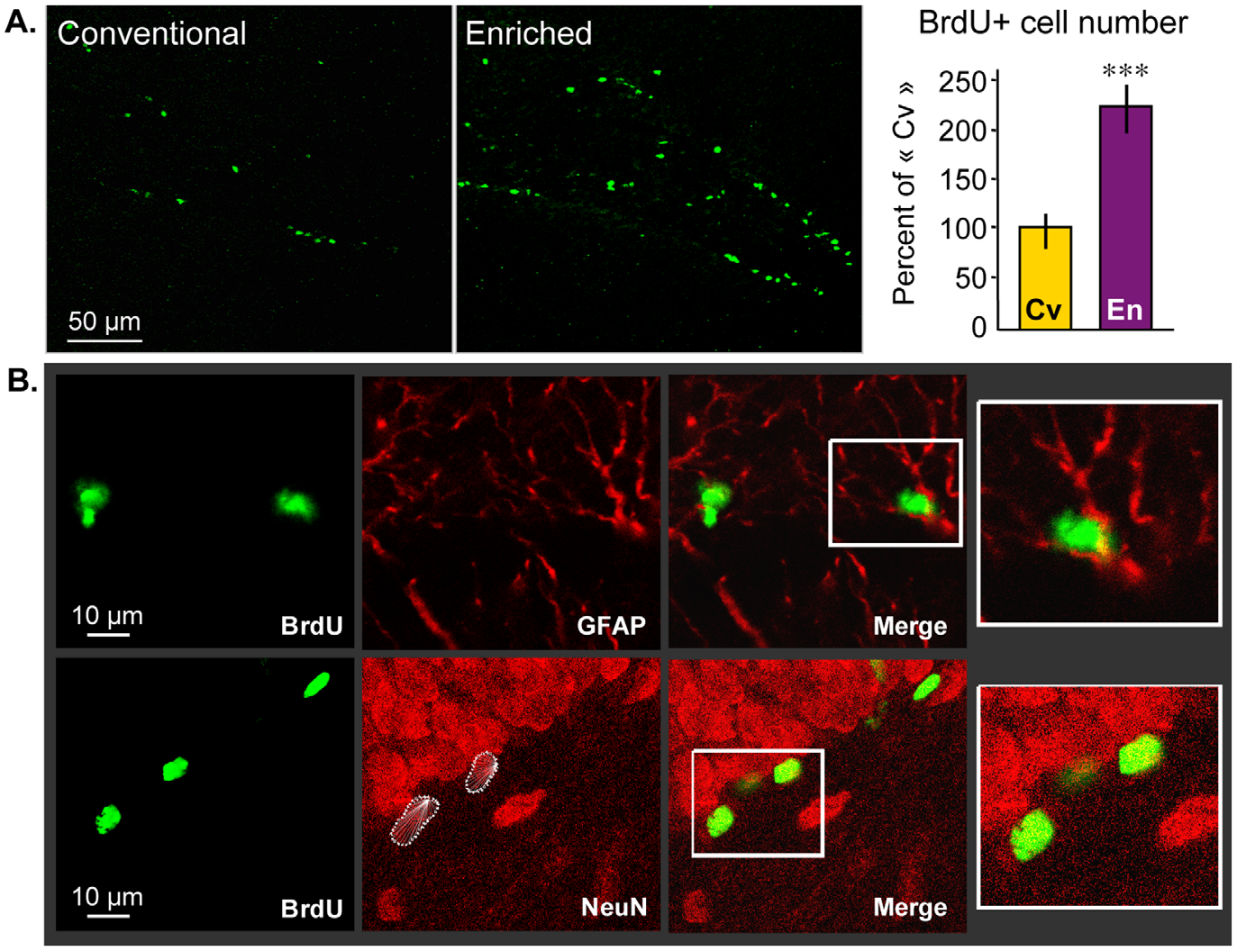
Marlau™ cages meet the criterion of enrichment-induced hippocampal neurogenesis. Fourteen days after BrdU injections, the averaged number of cells having incorporated BrdU (counted in 3 sections, 300 µm apart) nearly doubled in the hippocampus of rats raised in Marlau™ cages. While BrdU could be detected in both neurons and astrocytes, respectively identified by NeuN and GFAP, BrdU was primarily found in neurons. En vs. Cv: *** p<0.001, two-way ANOVA, factor 1: rat group, factor 2: anatomical plane. Each bar represents the mean ± SEM (*n = 4* in each group). Abbreviations: Cv and En as in Fig. 1.

As reported previously, environmental enrichment (EE) has been shown to increase neurogenesis in rodents [19], [20]. Here, we show fourteen days after 5′-bromodeoxyuridine (BrdU) administration that rats raised in Marlau™ cages had a greater number of BrdU-positive cells in the granule cell layer of the dentate gyrus (Fig. 4A). A substantial number of BrdU-positive cells in the dentate gyrus has been shown to differentiate into astrocytes (15–19%) following exposure to EE [19]. Our pilot studies showed that double immunolabeled BrdU-positive/NeuN-positive cells accounted for >90% of total BrdU-positive cells, and BrdU-positive/GFAP-positive cells with morphological features of mature astrocytes were rarely observed. These observations indicate that surviving BrdU-positive cells in our study differentiated primarily into neurons (Fig. 4B).

### Transcript Level of Genes Involved in Brain Plasticity

**Figure 5:**
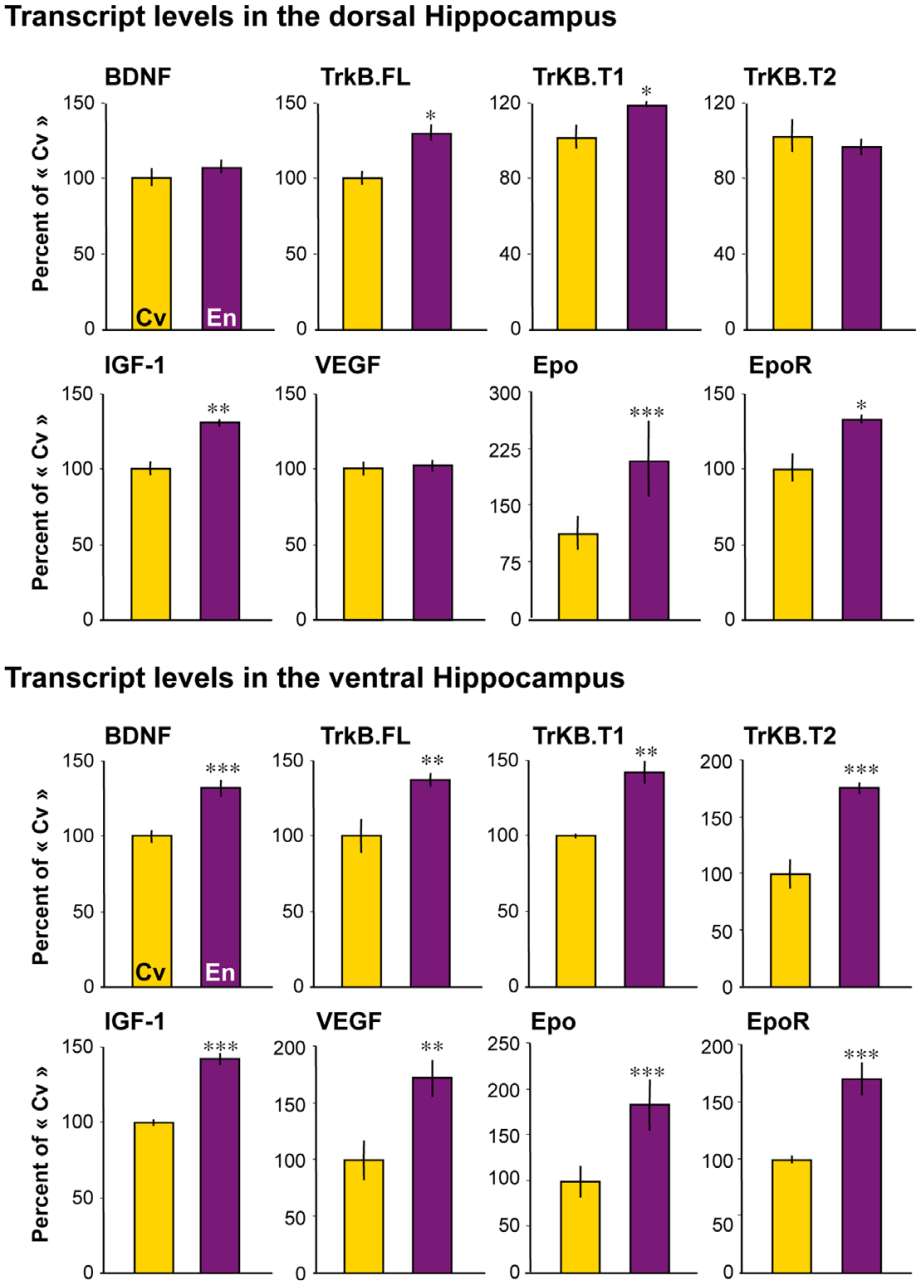
Marlau™ cages meet the criterion of enrichment-induced increase in transcript levels in the hippocampus. Enrichment-induced alterations in transcript levels were greater in the ventral hippocampus than in the dorsal hippocampus, for all genes measured after 6 weeks of housing in Marlau™ cages. En vs. Cv: * p<0.05, ** p<0.01, *** p<0.001, following two-way ANOVA, factor 1: rat group, factor 2: hippocampal subregion. Each bar represents the mean ± SEM (*n = 8* in each group). Abbreviations: Cv and En as in Fig. 1.

Effects of EE on brain plasticity are likely sustained by modulation of the expression patterns of many different genes [21]. Among these genes, those coding for Vascular Endothelial Growth Factor (VEGF) and Brain-Derived Neurotrophic Factor (BDNF) have been shown to play major roles in enrichment-induced neurogenesis [22], [23], and VEGF and BDNF transcript levels are induced in the hippocampus following EE [22], [24], [25]. Here, we show that BDNF and VEGF transcript levels were significantly induced in the ventral hippocampus, but not in the dorsal hippocampus of “enriched” rats compared to “conventional” rats (table 1; Fig. 5). BDNF exerts its function via full-length tyrosine kinase receptor TrkB.FL [26], and the truncated receptors TrkB.T1 and TrkB.T2 may regulate the local concentration of BDNF for extended periods of time following its endocytosis and release by astrocytes [27]. We show that transcript levels of TrkB.FL, TrkB.T1 and TrkB.T2 were significantly higher in the ventral hippocampus of “enriched” rats, compared to “conventional” rats. By contrast, TrkB.FL and TrkB.T1, but not TrkB.T2, transcript levels were significantly higher in the dorsal hippocampus of “enriched” rats (table 1; Fig. 5).

Erythropoietin (Epo) is an endogenous neuroprotective molecule [28], [29], and is involved in the regulation of a variety of physiological processes, including neurogenesis [30], [31] and dendritic and axonal outgrowth [32], [33], [34]. Interestingly, exogenous Epo induces BDNF transcript expression in the neocortex [35]. Here, we show that rats raised in Marlau™ cages had greater transcript levels of Epo and Epo receptor (EpoR) in the dorsal and ventral hippocampus (table 1; Fig. 5).

Compelling studies have provided evidence that Insulin-like Growth Factor 1 (IGF-1) works in concert with VEGF and BDNF to produce complementary effects of exercise on brain plasticity [36]. Hippocampal upregulation of IGF-1 transcript level has been reported after voluntary [37], but not forced [38], exercise. Because voluntary exercise is one of the behaviors promoted by the Marlau™ cage, we hypothesized that IGF-1 transcript level might be elevated in the hippocampus of rats raised in Marlau™ cages. In addition, exogenous IGF-1 induces transcript levels of Epo [39] and BDNF [38] in the hippocampus and the neocortex, respectively. Here, we show for the first time, to our knowledge, that IGF-1 transcript level was significantly increased in both the dorsal and ventral hippocampus of rats housed in the enriched environment compared to rats housed in conventional cages (table 1; Fig. 5).

Finally, transcript level of Microtubule-Associated Protein (MAP)-2 (table 1) was also significantly induced in rats raised in Marlau™ cages compared to rats raised in conventional cages, by +24±3.7% (p<0.05) and +60.8±15.0% (p<0.01) in the dorsal and ventral hippocampus, respectively, as previously reported by others for MAP-2 protein in the mouse hippocampus [40].

### Repeatability of the Results Obtained in Marlau™ Cages

**Figure 6:**
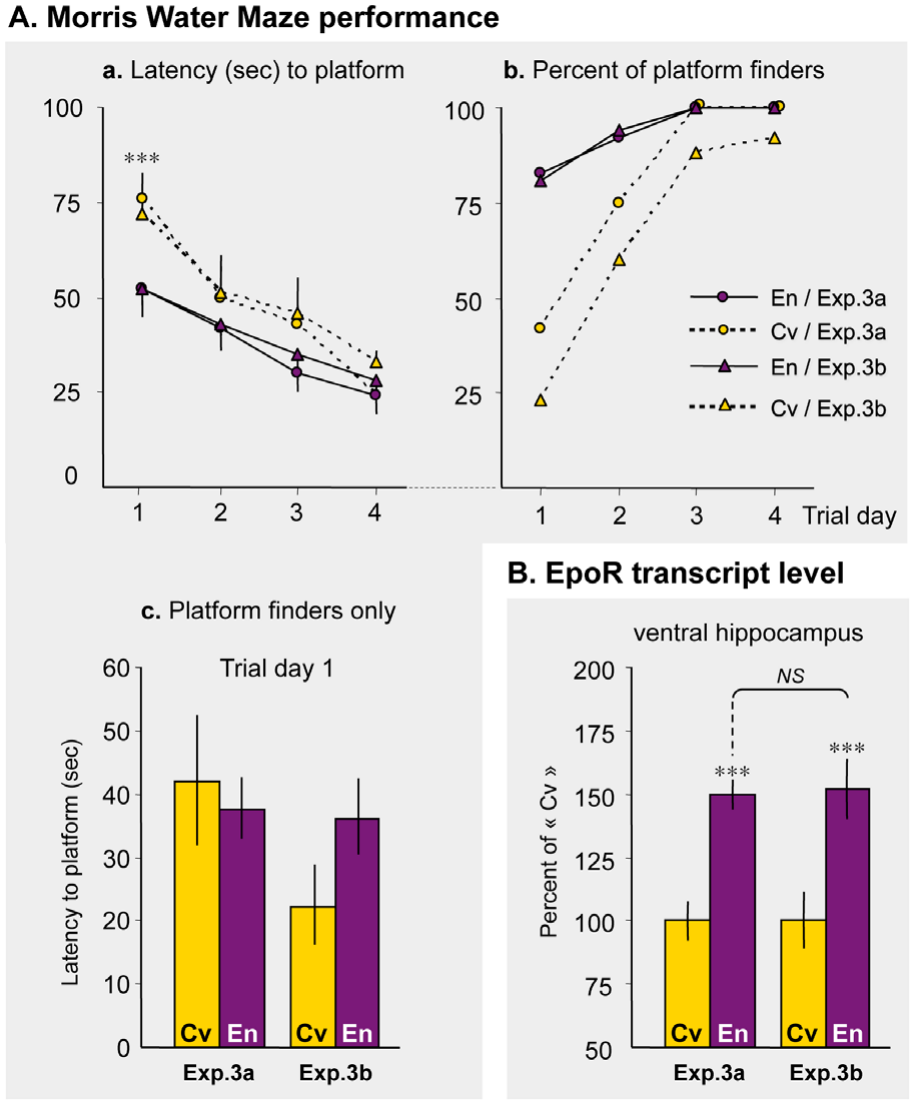
Marlau™ cages provide highly reproducible activation of hippocampal plasticity-related variables. Experiments were repeated twice (a and b), with 12 rats in each group. **A.** Six weeks after housing in Marlau™ or conventional cages, rats were tested four times a day for navigational/spatial learning in the Morris Water Maze. En vs. Cv: *** p<0.001, two-way repeated measures ANOVA. **B.** Two weeks later, EpoR transcript level was measured in the ventral hippocampus. En vs. Cv: *** p<0.001, two-way ANOVA. Abbreviations: Cv and En as in Fig. 1.

The hippocampal dentate gyrus plays an important role in learning and memory, in particular during navigational/spatial learning in the Morris Water Maze (MWM) test [41]. The improved performances in the MWM promoted by EE [42] have been confirmed half of the 36 studies listed in table 2. We investigated whether performances in the MWM were enhanced in rats raised in Marlau™ cages (Exp. 3a), and tested whether it could be replicated (Exp. 3b). We first determined the latency to find the platform as reported in the literature, and a maximum time (90 sec) was assigned to rats that did not find the platform. We report that similar results were obtained between experiments 3a and 3b, and that rats housed in Marlau™ cages performed better during the first trial day (Fig. 6A.a). However, this difference between “enriched” and “conventional” rats was the result of a greater proportion of “enriched” rats that found the platform on the first test day (Fig. 6A.b), and not the result of greater individual performances in “enriched” rats. That is, when rats found the platform on the first day trial, they performed similarly whether they were raised in Marlau™ cages or conventional cages (Fig. 6A.c). Similar results were obtained when the distance traveled was chosen as a variable (data not shown).

**Table 2:** Discrepancies in the effect of environmental enrichment on navigational/spatial- memory test (Morris water Maze test) in articles published during the 1993–2008 period in rodents is independent of the number of animals housed in each housing condition.

Equal number of animals between Cv and En housing conditions :		
Positive effects on spatial learning		
nb in Cv cages	nb in En cages	References
3	3	Schrijver N.C. 2002 Pharmacol Biochem Behav, 73∶209; Schrijver N.C. 2004 Behav Brain Res, 152∶307
4	4	Frick K.M. 2003 Neurobiol Aging, 24∶615
5	5	Bennett J.C. 2006 Neurobiol Learn Mem, 85∶139
**No effect on spatial learning**		
**nb in Cv cages**	**nb in En cages**	**References**
3–4	4	Gobbo O.L. 2005 Behav Brain Res, 159∶21
4–6	4–6	Boehm G.W. 1996 Brain Res, 726∶11
**Isolated animals in Cv conditions compared to “n” animals in En housing conditions:**		
**Positive effects on spatial learning**		
**nb in Cv cages**	**nb in En cages**	**References**
1	2	Holguin S. 2008 Behav Brain Res, 191∶11
1	6–8	Falkenberg T. 1992 Neurosci Lett, 138∶153; Guilarte T.R. 2003 Ann Neurol, 53∶50; Nilsson M. 1999 J Neurobiol, 39∶569; Pham T.M. 1999 Behav Brain Res, 103∶63; Toscano C.D. 2006 Exp Neurol, 200∶209
1	12	Galani R. 1997 Neurobiol Learn Mem, 67∶43
**No effect on spatial learning**		
**nb in Cv cages**	**nb in En cages**	**References**
1	6–8	Hicks R.R. 2002 Neuroscience, 112∶631; Puurunen K. 1997 Stroke, 28∶623; Schneider J.S. 2001 Brain Res, 896∶48
1	12	Wainwright P.E. 1993 Neurotoxicol Teratol, 15∶11
**Unequal number of animals between Cv and En housing conditions:**		
**Positive effects on spatial learning**		
**nb in Cv cages**	**nb in En cages**	**References**
2	8–12	Bredy T.W. 2003 Neuroscience, 118∶571; Hellemans K.G. 2004 Dev Brain Res, 150∶103; Zanardi A. 2007 Faseb J, 21∶4028
3–4	8–16	Jankowsky J.L. 2005 J Neurosci, 25∶5217; Kempermann G. 1997 Nature, 386∶493; Meshi D. 2006 Nat Neurosci, 9∶729; Wolff M. 2008 Hippocampus, 18∶996
15	30	Foley A.G. 2005 J Neurosci Res, 82∶245
**No effect on spatial learning**		
**nb in Cv cages**	**nb in En cages**	**References**
2–3	5–10	Briones T.L. 2006 Exp Neurol, 198∶530, Behav Brain Res, 168∶261; Cao X. 2008 Dev Psychobiol, 50∶307; Leggio M.G. 2005 Behav Brain Res, 163∶78; Mandolesi L. 2008 J Alzheimers Dis, 15∶11; Martinez-Cue C. 2005 Behav Brain Res, 163∶174; Ueda S. 2005 Neuroscience, 135∶395; van Rijzingen I.M. 1997 Neurobiol Learn Mem, 67∶21; Wang C.A. 2007 Epilepsy Behav, 11∶303
3–4	4–8	Comeau W. 2008 Dev Psychobiol, 50∶134; Miu A.C. 2006 Behav Brain Res, 172∶135; Wurm F. 2007 Stroke, 38∶2833

The transcript level of EpoR in the ventral hippocampus was chosen as another variable to test the repeatability of the results obtained in Marlau™ cages, since we recently demonstrated that the efficacy of exogenous Epo to protect neurons against excitotoxic injury was determined by the expression of EpoR in brain tissue [43]. Here, we report that the difference in EpoR transcript level measured in the ventral hippocampus of rats raised in Marlau™ cages and conventional cages was similar between experiments 3a and 3b (Fig. 6B).

### Synaptic Plasticity in CA1 Area of the Hippocampus

**Figure 7:**
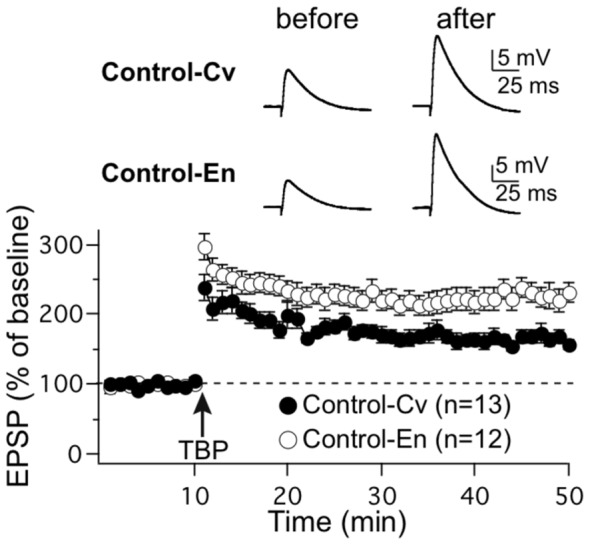
Rats raised in Marlau™ cages displayed increased LTP in CA1 area neurons. LTP elicited by TBP increased significantly in rats housed in Marlau™ cages compared to rats housed in conventional cages (n = 13 in Cv group; n = 12 in En group). Each data point represents the average of three successive test responses evoked at 0.05 Hz. The mean slope of the EPSP recorded 0–10 min before TBP was taken as 100%. The top traces show EPSPs before and after LTP induction. Arrow marks the starting point of tetanus stimulation. Results are presented as the mean ± SEM (n = number of cells; 1–2 cell(s) per rat).

Synaptic gain change, which reflects the strength of neuronal responsiveness to synaptic activity, can result from modifications in properties of synaptic transmission such as following long-term potentiation (LTP) and long-term depression. In hippocampus, LTP is a well-established general candidate mechanism for learning and memory [44], [45]. Many studies have demonstrated in rats that facilitation of LTP in area CA1 of the hippocampus can be achieved after 3 to 8 weeks of continuous EE, using extracellular field potential recordings [46], [47], [48], [49]. A majority of these earlier studies, however, used extracellular field potential recordings. Here, we demonstrate, using whole-cell patch-clamp recordings in rat hippocampal slices, that theta-burst pairing (TBP)-induced LTP was facilitated in area CA1 of the hippocampus as soon as 1–2 weeks of EE in Marlau™ cages (Fig. 7). Indeed, we found that potentiation of EPSP amplitudes increased by 166.0±8.9% for rats housed in conventional cages (n = 13; t = 45−50 min after TBP, p = 0.0001) and by 227.0±14.4% for rats raised in Marlau™ cages (n = 12; t = 45−50 min after TBP, p = 0.0001), the difference in LTP amplitude between the two groups of rats being highly significant (p = 0.0013).

### Water Exploration Test as a Reliable Way to Measure Anxiety in All Rats

**Figure 8:**
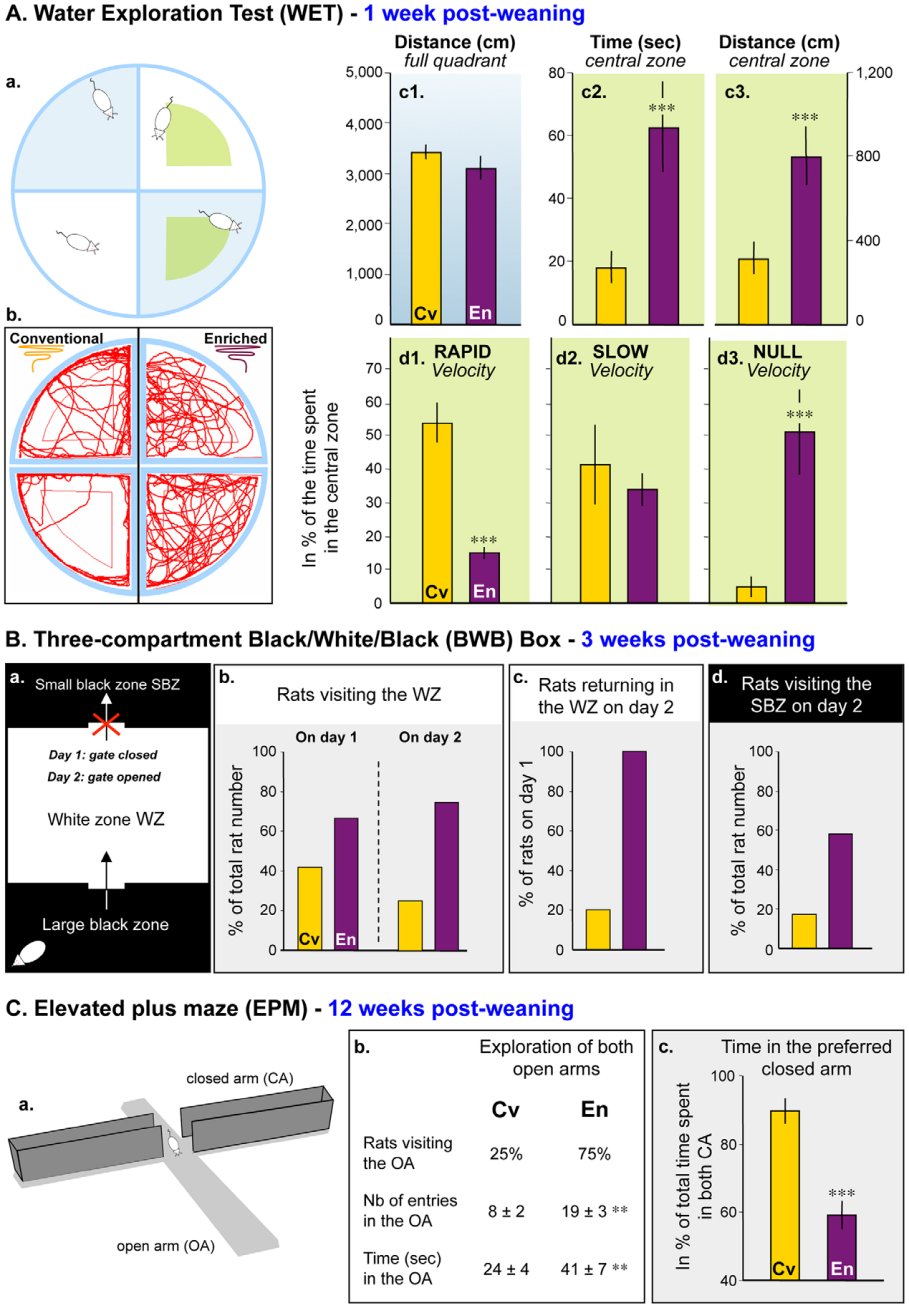
Longitudinal analysis of exploratory behavior during housing in Marlau™ cages is in favor of decreased anxiety in “enriched” rats. Rats were consecutively tested in the WET (A), in the BWB (B), and in the EPM (C), respectively after one, three and twelve weeks of housing in Marlau™ or conventional cages. **A.** WET. *a,* rats are placed in a pool divided into 4 quadrants for 5 min; visiting the virtual “central zone” (CZ, green) was indicative of reduced anxiety; *b,* typical trajectories of S and En rats; *c,* time spent and distance traveled in the full quadrant and the CZ; *d,* time spent in the CZ swimming with different velocity profiles: rapid, slow, and null (floating behavior). Each bar represents the mean ± SEM (*n = 12* in each group). **B.** BWB. *a,* rats were tested during 2 consecutive days (with access to the SBZ on day 2 only); visiting the WZ on day 1 and the SBZ on day 2 was indicative of reduced anxiety; *b-d,* we measured the number of rats visiting the WZ on days 1 and 2 (*b*), the percentage of those returning to the WZ on day 2 (*c*), and the number of rats visiting the SBZ on day 2 (*d*). Each bar represents the mean of the group (n = 12 in each group). **C.** EPM. *a*, exploratory behavior of rats was tested for 5 min once placed in an open arm (OA), as shown; exploration of the OA was indicative of reduced anxiety; *b,* we measured the number of rats visiting the OA, the number of entries in the OA, and the time spent in the OA; *c,* while the time spent to explore the closed arms (CA) was mostly restricted to one arm in “conventional” rats, the “enriched” rats explored nearly equally both CA. En vs. Cv: ** p<0.01, *** p<0.001, Student’s *t* test. Abbreviations: Cv and En as in Fig. 1.

Marlau™ cages include a series of branching mazes and multiple compartments. Since most of the tests available to measure anxiety-like behavior comprise either different branches or at least two compartments (EPM, dark/light box), experimental bias may be caused when comparing the level of anxiety of rats raised in Marlau™ cages compared with that of rats raised in conventional cages. Therefore, we developed the Water Exploration Test (WET) as a new tool to measure anxiety, as shown in Fig. 8A.a. Rats were allowed to explore a quadrant of a circular pool for 5 min; the central zone of the quadrant was referred to as the anxiogenic zone. The swimming distance in the full quadrant, the time spent, the swimming distance and the velocity in the central zone of the quadrant were measured in rats of 9 weeks of age, administered intraperitoneally either with saline, the anxiogenic drug FG-7142 (25 mg/kg), or the anxiolytic drug diazepam (3 mg/kg) 30 min prior to WET. A reduction of the time spent in the central zone and of the time spent at null velocity (floating behavior) in this zone is indicative of an anxiety-like behavior, which is consistent with results obtained in “conventional” rats treated with FG-7142 or diazepam (table 3). The anxiogenic effect of FG-7142 was even better demonstrated in “enriched” rats (table 4).

**Table 3:** Effect of FG-7142 (25 mg/kg, i.p.) and diazepam (3 mg/kg; i.p.) treatments, administered 30 min prior to WET in Sprague-Dawley rats at 9 weeks of age, raised in conventional cages.

	Full Quadrant	Central Zone				
	Total distance (cm)	Total distance (cm)	Time (sec)	Rapid velocity (sec)	Slow velocity (sec)	Null velocity (sec)
Controls	3,735±132	1,189±82	24.6±2.7	8.0±1.4	10.3±1.0	6.3±0.9
FG-7142	3,872±179	988±121	17.5±2.0	7.3±0.6	6.9±0.8	3.3±0.3
*% of controls*	*104±5*	*83±12*	*71±8* *	*91±7.5*	*67±8* *	*52±5* **
Diazepam	3,772±112	1,224±132	34.2±2.9	8.7±0.6	13.6±1.3	11.9±0.9
*% of controls*	*101±3*	*103±11*	*131±12* *	*109±8*	*132.0±13*	*189±15* ***

Differences between controls and FG-7142 or diazepam-treated rats:

* p<0.05;

** p<0.01;

*** p<0.001 (ANOVA 1).

**Table 4:** Effect of FG-7142 (25 mg/kg, i.p.) treatment, administered 30 min prior to WET in Sprague-Dawley rats at 9 weeks of age and raised in Marlau™ cages for 6 weeks.

	Full Quadrant	Central Zone				
	Total distance (cm)	Total distance (cm)	Time (sec)	Rapid velocity (sec)	Slow velocity (sec)	Null velocity (sec)
Controls	3,986±98	1,869±115	41.5±2.8	7.0±0.6	18.6±1.7	15.9±1.3
β-Carboline	4,667±232	1,149±122	21.5±2.3	15.5±1.9	3.0±0.6	3.0±0.6
*% of controls*	*117±6* *	*61±7* ***	*52±6* ***	*223±27* ***	*16±3* ***	*19±4* ***

Differences between controls and FG-7142:

* p<0.05;

** p<0.01;

*** p<0.001 (ANOVA 1).

### Longitudinal Analysis of Exploratory Behavior

One week after weaning and housing in Marlau™ cages or conventional cages, the behavior of the rats was tested in the WET, as explained above. The path (Fig. 8A.b), the swimming distance in the full quadrant (Fig. 8A.c1), the time spent (Fig. 8A.c2) and the swimming distance (Fig. 8A.c3) in the central zone of the quadrant, and the velocity in the central zone (Fig. 8A.d1–3) were measured. “Enriched” and “conventional” rats had different exploration patterns. Whereas rats in both groups swam the same distance (Fig. 8A.c1), “enriched” rats explored the central zone more thoroughly (Fig. 8A.b, c2, c3). In addition, when present in the central zone, “enriched” rats adopted a floating behavior (null velocity, Fig. 8A.d3), while “conventional” rats just crossed it rapidly (rapid velocity, Fig. 8A.d1).

After 3 weeks of housing in Marlau™ cages or conventional cages, rat behavior was tested for 90 sec during two consecutive days in the three-compartment Black/White/Black (BWB) box, as described (Fig. 8B.a). Visiting the white zone was indicative of less anxiety. On the first day, when rats had access to 2 compartments only and were introduced into the large black zone, the adjacent white zone was more frequently visited by rats of the enriched group than by conventionally housed rats (Fig. 8B.b). On the second day, when rats were introduced in the large black zone, and were allowed to access a small black zone after crossing the white zone, fewer conventionally housed rats revisited the white zone on the second day than “enriched” rats. Conversely, all rats of the enriched group revisited the white zone on the second day (Fig. 8B.c), and the majority of them explored the small black zone (Fig. 8B.d).

After 12 weeks of housing in conventional or Marlau™ cages, rat behavior was tested for 5 min in the EPM (Fig. 8C.a). The proportion of rats visiting the open arms was three-fold greater in the enriched group than in the conventional group, and the number of entries and the time spent in the open arms were significantly greater in the enriched group (Fig. 8C.b). In addition, analysis of the exploratory behavior in the closed arms revealed that rats in the conventional group spent most of the time in one arm (the “preferred closed arm”) than in the other, while rats in the enriched group spent about half of the time in each closed arm (Fig. 8C.c).

### Cognitive Performances at Different Stages Following *Status Epilepticus* in Rats

**Figure 9:**
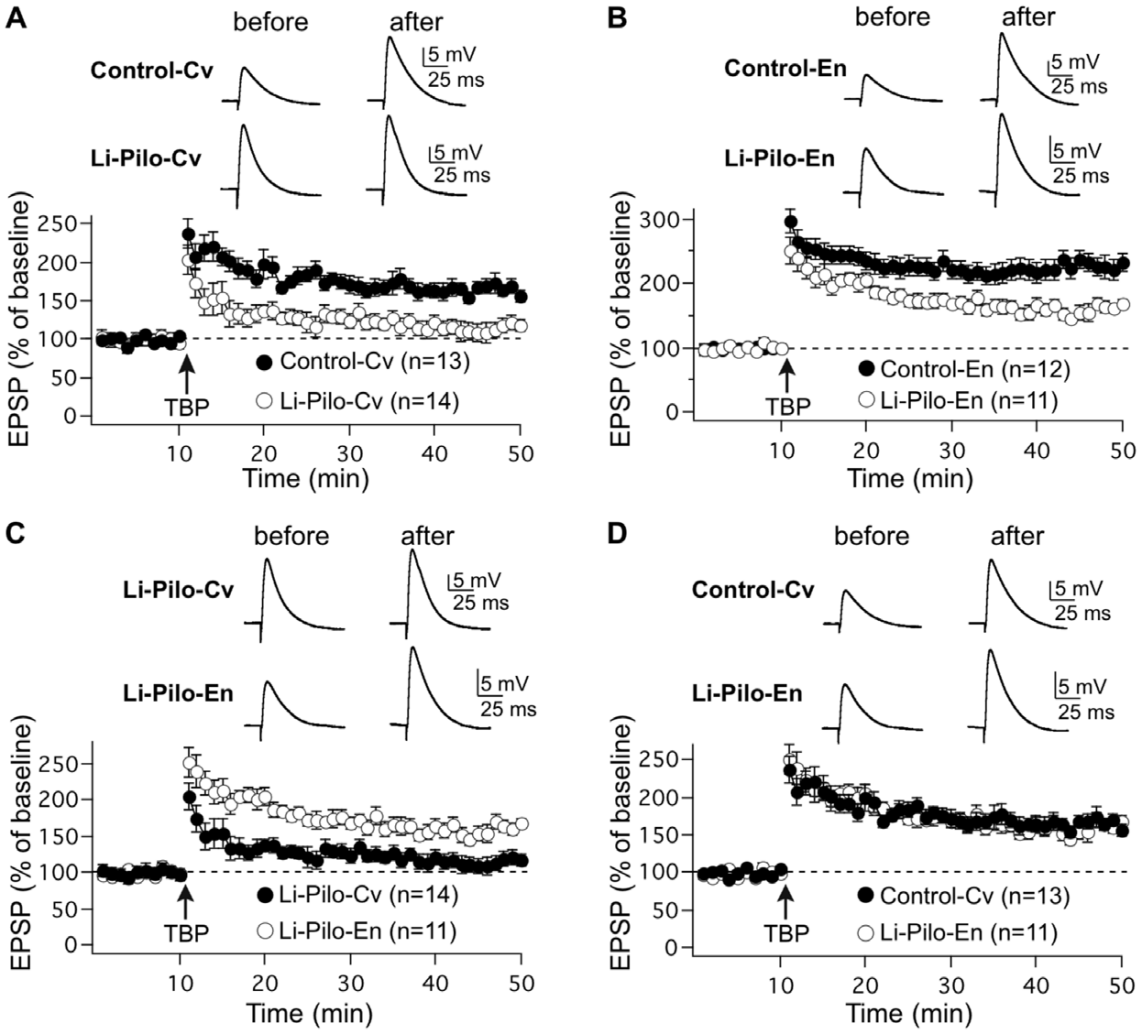
EE in Marlau™ cages immediately after Li-Pilo-SE at weaning improves LTP in CA1 pyramidal neurons observed 1–2 weeks later after SE. **A.** Induction of LTP in CA1 neurons was significantly (p = 0.0003) impaired in slices of rats housed in conventional cages following Li-Pilo-SE (n = 14) compared with healthy rats housed in the same cages (n = 13). **B.** Induction of LTP in CA1 neurons was significantly decreased (p = 0.0006) in slices of rats housed in Marlau™ cages following Li-Pilo-SE (n = 11) compared with healthy rats housed in the same cages (n = 12). **C.** LTP in CA1 pyramidal neurons of rats subjected to Li-Pilo-SE at weaning was significantly higher (p = 0.0003) when rats were housed in Marlau™ cages (n = 11) compared to those housed in conventional cages (n = 14). **D.** Induction of LTP in rats subjected to Li-Pilo-SE and then housed in Marlau™ cages (n = 11) was statistically similar (p = 0.98) to that of healthy rats housed in conventional cages (n = 13). The top traces show EPSPs before and after LTP induction. Arrow marks the starting point of tetanus stimulation. Results are presented as the mean ± SEM (n = number of cells; 1–2 cell(s) per rat). Abbreviations: Cv and En as in Fig. 1.

Prior studies revealed that spatial/navigational learning and memory in the MWM was dramatically altered in rats 4 weeks after the induction of *status epilepticus* by lithium-pilocarpine (Pilo-SE) at weaning [50], [51], [52]. Our first objective was to determine whether cognitive deficits could be observed earlier following SE. However, it is barely possible to test learning and memory in the MWM 1–2 weeks after Pilo-SE in immature rats. During that period, we thus assessed LTP in CA1 pyramidal neurons, a cellular mechanism underlying learning and memory in the hippocampus [44], [45]. We show that LTP monitored 1–2 weeks after Pilo-SE in rats housed in conventional cages (115.5±9.3%, n = 14, t = 45−50 min after TBP, p = 0.028; Fig. 9A) was significantly reduced compared to healthy controls housed in the same cages (p = 0.0003; Fig. 9A).

Prior studies revealed that EE immediately after Pilo-SE induced at weaning partially preserved spatial/navigational learning and memory [50], [51], [52]. Our data demonstrate that 1–2 weeks of housing in Marlau™ cages was sufficient to improve LTP in healthy rats (Fig. 7). Therefore, we examined whether 1–2 weeks of EE in Marlau™ cages can help to prevent the deterioration of LTP induction observed in rats subjected to Pilo-SE at weaning. We show that the magnitude of LTP monitored in rats subjected to Pilo-SE and housed in Marlau™ cages (162.7±7.5%, n = 11, t = 45−50 min after TBP, p = 0.00001; Fig. 9B) was significantly reduced compared to healthy controls housed in the same cages (p = 0.0006; Fig. 9B), but was significantly improved compared to rats subjected to Pilo-SE housed in conventional cages (p = 0.0003; Fig. 9C). Intriguingly, LTP amplitude of rats subjected to Pilo-SE at weaning and then housed in Marlau™ cages was similar to that of healthy rats of the same age housed in conventional cages (p = 0.98; Fig. 9D). Altogether, our data demonstrate that cellular mechanisms underlying learning and memory in the hippocampus can be altered during epileptogenesis, a deficit that can be counteracted by housing in Marlau™ cages.

**Figure 10:**
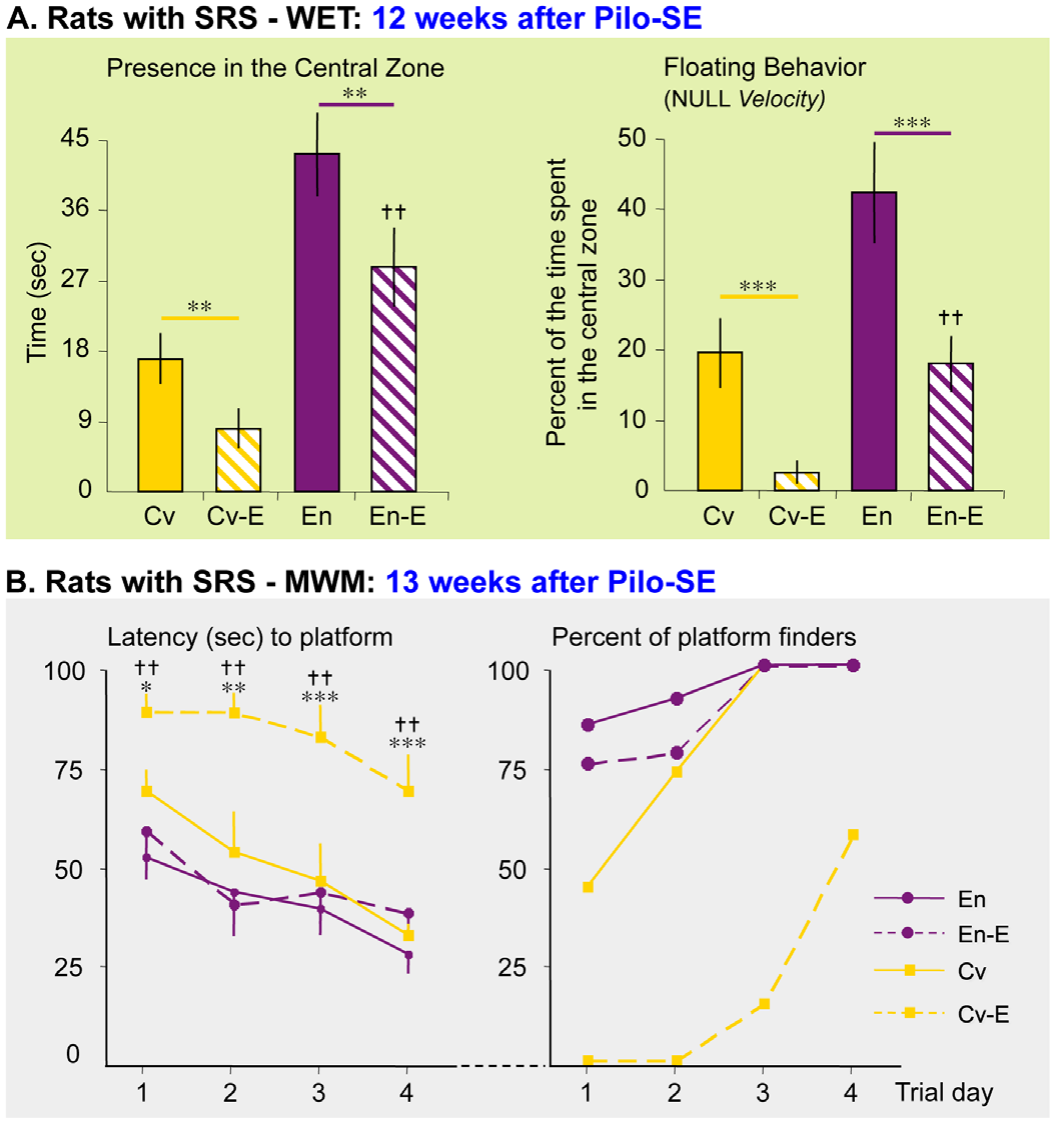
Rats raised in Marlau™ cages after excitotoxic brain injury displayed decreased cognitive impairments. Rats underwent pilocarpine-induced *status epilepticus* (Pilo-SE) at 3 week-old, and were raised, on the following day, either in conventional or Marlau™ cages. Rats developing spontaneous recurrent seizures (SRS, or “epileptic”) were then subjected at 15- and 16-week old to the WET and to the MWM, respectively. In these results, 10 rats not subjected to Pilo-SE were included in each Cv and En groups. The number of rats subjected to Pilo-SE that developed SRS was not identical in each Cv (n = 16/18) and En (n = 9/18) groups. **A.** WET. While rats undergoing SRS displayed marked anxiety-like behavior, the positive effect of enrichment in control rats was observed in epileptic rats, as demonstrated by the increased time spent floating in the central zone. Cv-E vs. Cv and En-E vs. En: ** p<0.01, *** p<0.01; En-E vs. Cv-E: 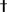 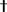
p<0.01, ANOVA 2. **B.** MWM. Enriched housing prevented the deterioration of learning and memory observed in conventional housing, as indicated by the latency to find the platform and the proportion of rats finding the platform. Cv-E vs. Cv: * p<0.05, ** p<0.01, *** p<0.01; En-E vs. Cv-E: 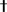 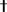
p<0.01, two-way repeated measures ANOVA. Abbreviations: Cv and En as in Fig. 1; En-E, epileptic rats housed in enriched Marlau™ cages; Cv-E, epileptic rats housed in conventional cages.

To our knowledge, no study has explored the effect of housing on the cognitive function of adult epileptic animals that had been subjected to Pilo-SE at weaning. In this study, it was not possible to perform continuous EEG monitoring in rats housed in social groups, in particular within the Marlau™ cages that present a complex three-dimensional structure. Our setup did not allow us to monitor individual movements in all compartments of the Marlau™ cage during the dark phase; thus two persons observed the rats during 8 hrs a day, 5 days a week, from week 10 to week 13 post-SE. At the end of the 11^th^ week after Pilo-SE, 16 out of 18 rats raised in conventional cages and 9 of 18 rats raised in Marlau cages exhibited spontaneous recurrent seizures (SRS) during the light phase, suggesting that housing in enriched Marlau™ cages reduced the proportion of rats developing SRS after Pilo-SE (p = 0.0113, Person’s Chi^2^ test). However, we cannot guarantee that rats free of spontaneous seizures during the light phase did not have seizures during the dark phase. Because our objective was to determine whether enriched environment can reduce the severity of comorbid anxiety and cognitive deficits in rats with spontaneous seizures, only rats that exhibited SRS by the end of the 11^th^ week after Pilo-SE were tested for anxiety and spatial learning performances. Anxiety has been shown to increase in rats raised in conventional housing conditions after kainic acid-induced SE, this effect being reversed by housing in non-standardized enriched environments [53]. In our study, all rats with SRS displayed increased anxiety-like behavior compared to respective controls (conventional or Marlau™ cages). However, anxiety-like behaviors in rats with SRS raised in Marlau™ cages were far below that measured in rats with SRS raised in conventional cages (Fig. 10A).

Non-standardized EE has already been shown to protect navigational/spatial memory in the Morris Water Maze of rats subjected to Pilo-SE, either partially [51] or fully [52]. However, in the latter study, no difference was observed in the latency to find the submerged platform between conventional and enriched control groups [52]. Here we show that performances in the Morris Water Maze test were highly protected in epileptic rats raised in Marlau™ cages. Indeed, while none of the epileptic rats raised in conventional cages found the platform during the first two trial days, most of the rats (>75%) that were raised in Marlau™ found the platform on the first trial day. In general, epileptic rats raised in Marlau™ cages displayed memory performances similar to control “enriched” rats (Fig. 10B).

## Discussion

The development of the Marlau™ cage provides a standardized enrichment procedure for the housing of rodents for preclinical research studies. We engineered the Marlau™ cage for this purpose, and the findings of this study demonstrate that the Marlau™ cage offers reduced stressful social interactions, increased voluntary exercise, diverse entertainment activities, and standardized cognitive stimulation and novelty. Compared to rats raised in conventional cages, rats raised in Marlau™ cages exhibited the phenotypic characteristics reported for animals housed in non-standardized EE, i.e. increased (i) cortical thickness, (ii) hippocampal neurogenesis, (iii) hippocampal levels of BDNF and VEGF transcripts, better performances in spatial learning, and decreased anxiety-trait. In addition, LTP was increased in rats housed in Marlau™ cages as soon as one week after exposure. Finally, housing rats in Marlau™ cages after severe *status epilepticus* at weaning prevented subsequent cognitive impairments associated with seizure-induced brain damage.

### Development of the Water Exploration Test to Measure Anxiety

Most of the tools available to measure anxiety in laboratory animals, and in rodents in particular, are composed of branching mazes. Rats raised in Marlau™ cages are living in an environment that includes a series of branching mazes. Therefore, to circumvent any potential bias when comparing their level of anxiety with that of rats unacquainted with branching mazes, we developed the WET as a new tool to measure anxiety. Using the anxiogenic drug FG-7142 [54] and the anxiolytic drug diazepam [55], we determined that increase (i) in the time spent in the central zone of the quadrant and (ii) in the percentage of time spent with a floating behavior in this central zone were indicative of an anxiety-like behavior in healthy Sprague-Dawley rats. It is noteworthy that when these two variables were measured one week or twelve weeks after weaning, we found that they were always greater in rats housed in Marlau™ cages compared to rats housed in conventional cages. This finding indicates that Sprague-Dawley rats raised in enriched Marlau™ cages exhibit lower anxiety-like behavior compared to rats raised in conventional cages.

The Elevated Plus Maze (EPM) belongs to the family of the branching mazes. It comprises four arms and is widely used to measure anxiety in rodents. It is noteworthy that twelve weeks after weaning, two independent studies using either the EPM or the WET concluded that anxiety was reduced in rats raised in Marlau™ cages compared to rats housed in conventional cages. However, we noticed that the WET presents an important advantage over the EPM when testing anxiety in rodents. In the WET, rats have to swim continuously. By contrast, in the EPM, absence of motivation to explore the maze can lead to an ambiguous interpretation of the time spent in the open arms.

### The Marlau™ Cage Fulfills Both Research and Animal Welfare Objectives

A distinction has been made between enrichment protocols used in neuroscience research and in animal welfare research [56]. In neuroscience research, EE includes increased social and sensory stimulations, and it has long been used to evoke brain plasticity or to produce greater recovery after brain insults or resistance in drug addiction [2], [3], [10]. Usually, enriched protocols consist in housing animals in large cages with toys that are changed frequently to induce changes in brain and behavior. By contrast, the main focus in animal welfare research is to improve the wellbeing of animals by allowing the development of species-specific behavior. To meet the principles of the two research fields, we developed the enriched Marlau™ cage.

In the wild, rats are constantly facing problems that must be overcome in order to survive and thrive, and they do so by using their complex range of cognitive skills [57]. We addressed this characteristic in the Marlau™ cage by making food acquisition a critical factor in survival. Food and water are not easily within reach in the Marlau™ cage. When rodents are in the compartment comprising water bottles, they are separated from the food pellet compartment by a maze that increases the difficulty to acquire food. Complexity is reinforced at every maze configuration change, since animals have to find a new path through the maze three times a week, i.e. at every maze change, they must use cognitive processes including learning and memory.

The change in maze configuration was inspired by the training program in the original “environmental complexity and training” paradigm [58]. In ECT training, rodents were subjected to daily exploration of a maze with frequent changes in the pattern of barriers. The major advantage of the Marlau™ cage compared to the ECT program is that the maze is a permanent constituent of the Marlau™ cage, while daily trainings in the ECT program were performed in cages distinct from the housing cages.

In the literature, there is no consensus on the number of animals that should be housed in control (conventional) cages to discern the effects of enriched housing using larger groups of animals. For a considered variable (i.e. navigational/spatial-memory test in the Morris Water Maze), the data in table 2 clearly show that the number of animals housed in each environment is not a key parameter that can explain the inter-experiment variability. Moreover, some attempts had been initiated to determine what component in the enriched environment was responsible for the beneficial effects on brain plasticity. While voluntary exercise has been reported to be a major component [59], it is not the case for social housing. Indeed, social grouping cannot account for cerebral effects of enriched environments [1]. Thus, it is very unlikely that the effects observed in our study between conventional and enriched Marlau™ cages are due to a variation in the number of animals (6 and 12 rats per cage in conventional and Marlau™ cages, respectively).

The design of the Marlau™ cage makes it possible for animals to feel a certain level of control over the environment, by making decisions and by facilitating escape behavior from a dominant animal. These features, which all contribute to increased wellbeing [57], may explain our data showing that rats raised in Marlau™ cages had a greater capacity to cope with stress, as demonstrated by the faster recovery of basal plasma CORT levels in response to restraint stress, compared to rats raised in conventional cages. These results, which are consistent with prior results obtained by others in mice [60], may be partly explained by the increased expression of glucocorticoid receptors in the hippocampus of rats raised in Marlau™ cages, given that these receptors contribute to the rapid recovery of basal plasma CORT levels following acute stress [15].

Regular maze change configuration in the Marlau™ cage generates complexity and novelty, two crucial features of EE in neuroscience research. Maze change configuration also offers to rodents the opportunity to develop species-specific behaviors, especially when challenged by critical factors in survival. This feature may primarily contribute to wellbeing when animals possess the skills to overcome the challenge [57]. The Marlau™ cage thus fulfills neuroscience research and animal welfare objectives.

### Marlau™ Cage Provides Equal Exposure to Challenge and Homogenized Results

Due to the location of the maze separating water and food compartments, the Marlau™ cage presents two main advantages. First, the challenge that consists in navigating through the maze to acquire food is affecting all animals similarly. Second, the challenge is such that all rats possess the skills to overcome it. Equal exposure to the food-searching challenge was verified by body weight measurements. Indeed, while contrasting with a recent study performed on Sprague-Dawley rats housed in non-standardized EE [61], we did not find any decrease in the body weight of any rats housed in Marlau™ cages, as already reported for diverse mouse strains [62], [63], [64], [65]. The weight gain was associated with stable total body fat percentage, indicating that the general growth of rats raised in the Marlau™ cage was enhanced compared to rats raised in conventional cages.

Concerns have been raised that enriched housing might disrupt standardization and thus affect the reproducibility of results when measuring biological and biochemical variables or in behavioral tests. However, a study involving three laboratories with standardized cages, enrichment protocol, light phase, test system and test protocol across laboratories has demonstrated that variance in the results obtained was not significantly higher between laboratories than between replicates in each laboratory [66]. This effect of EE contrasts with the lack of reproducibility that has been noted between laboratories when animals were housed in conventional cages, despite a high degree of standardization of the procedures and protocols between laboratories [67]. Nevertheless, although EE paradigms reduce variability in replicates within a same laboratory and between laboratories using exactly the same procedures [66], significant inconsistencies have been observed between numerous studies that looked at a same variable, i.e. the individual performance in spatial learning and memory, when using different protocols of EE (table 2). Discrepancies between labs are difficult to explain. A lack of detailed descriptions of the enrichment protocols used in different publications [2], a pronounced variability in the enrichment protocols used by different investigators [68] and the possibility that all animals housed together are not exposed equally to the features that characterize a given EE protocol may be other sources of variability between studies.

We found that rats raised in Marlau™ cages performed better in the Morris Water Maze than rats raised in conventional cages, a result consistent with 50% of the studies analyzed (table 2). However, when looking at individual performances, we noticed that a minority of rats raised in conventional cages (1 to 2 in each cage of 6) was doing as well as rats raised in Marlau™ cages. Such good performers in conventional cages might correspond to dominant rats, as previously observed in CD-1 mice [69]. One of the most prevalent challenges for dominant rodents is to maintain social competition over subordinates. Such a competition might also exist in the Marlau™ cage. However, due to the large space areas and the presence of shelters in Marlau™ cages, social dominance might not impact animals as it does in conventional cages. If dominant rats in conventional cages really do perform as well as the majority of rats in the Marlau™ cage, we might have to associate good scores in the MWM with the ability of the rats to overcome challenges in their environment. When rats are housed in boring cages, the only challenge to overcome may be social competition. Thus, standardized procedures of EE in the Marlau™ cage might help to optimize homogeneity among animals, because they all overcome one of the most challenging issues in survival: food acquisition.

### Gene Expression and Neurogenesis in EE

BDNF has been considered a hallmark variable induced by exposure to environmental enrichment [70]. As BDNF protein level has been reported to increase in the ventral hippocampus [70], we provide evidence here that housing in Marlau™ cages up-regulates BDNF-mRNA level in the ventral hippocampus, and confirm that BDNF-mRNA level remains stable in the dorsal hippocampus [42], [71]. Up-regulation of BDNF-mRNA level has been reported in the dorsal hippocampus only when animals were subjected both to environmental enrichment and to a spatial learning task, and when results were compared to those obtained in animals housed in isolated conditions [25], [42].

Up-regulation of BDNF has been suggested to mediate enhanced neurogenesis in the hippocampus of animals raised in environmental enrichment [23]. However, as mentioned above, BDNF gene expression remains stable in the dorsal hippocampus, where enhanced neurogenesis has been reported in animals raised in environmental enrichment [19], [20]. Other genes, such as IGF-1 and Epo, up-regulated in the dorsal hippocampus of rats raised in the Marlau™ cage, might mediate enhanced hippocampal neurogenesis [30], [31], [72], [73].

With regard to VEGF, its enhanced hippocampal expression has been shown to be crucial for the induced neurogenesis and performance in learning and memory tasks following environmental enrichment [22]. Despite our previous study showing increased VEGF-mRNA level in the whole hippocampus of rats raised in non-standardized environmental enrichment [24], we show here that the level of VEGF-mRNA increased in the ventral, but not the dorsal, hippocampus of rats raised in Marlau™ cages, compared to rats raised in conventional cages.

### Housing in Marlau™ Cages Protects Cognitive Function in Epileptic Rats

Environmental enrichment has been shown to have beneficial effects in animal models of neurodegenerative diseases (reviewed in [10], [74]), such as Alzheimer’s disease [75], [76], [77], Huntington’s disease [78], [79], [80], [81], [82], [83], [84], and Parkinsons’s disease [85], neurodevelopmental disorders such as Rett Syndrome [86], psychiatric disorders such as schizophrenia [87], [88], and in brain trauma [89]. Environmental enrichment has also been shown to delay the development of kindling [90], inhibit kainate-induced SE [91], to prevent cognitive decline during epileptogenesis in rats subjected to Pilo-SE at weaning [50], [51], [52], and to delay the onset of seizures in a mouse model of Huntington’s disease [92]. However, to our knowledge, the question of whether environmental enrichment can prevent or delay the development of chronic epilepsy has never been addressed [93]. Our data suggest that housing in enriched cages (Marlau™ cages) after Pilo-SE induced at weaning reduces the number of rats that exhibit spontaneous recurrent seizures (SRS) later in life. In addition, we show that rats with SRS housed in Marlau™ cages do not develop the extent of cognitive decline (evaluated in the MWM) observed in rats with SRS that are housed in conventional cages. This effect observed in the long term (13 weeks after Pilo-SE) in rats with SRS may be partly explained by the partial preservation of LTP observed after 1–2 weeks of housing in Marlau™ cages following Pilo-SE. Also, protection of place navigation in the MWM may be due to continuous activation of hippocampal function in Marlau™ cages. Indeed, place navigation in the MWM depends on the functionality of “place” cells, which are hippocampal neurons that identify or represent points in space in an environment [94]. In rats housed in conventional cages, seizures cause a decline in the precision and stability of “place” cells [95]. By contrast, in rats housed in Marlau™ cages, the functionality of “place cells” might have been protected, despite the presence of SRS, likely through the solicitation of “place cell” activity each time rats had to learn new spatial representation forms [96] that occurred at every change in maze configuration. This result is of particular importance for translational research, since place- and view-specific hippocampal activity has been reported in both humans and non-human primates [97], [98], [99].

### Persistence of Anxiety in Rats with SRS Raised in Marlau™ Cages

In the WET, healthy rats raised in Marlau™ cages exhibited a reduced level of anxiety-like behavior compared to their counterparts raised in conventional cages, as indicated by the increased time spent floating in the center of the quadrant. At first glance, this might indicate that rats housed in Marlau™ cages developed an inadequate behavior with respect to predator threat in the wild. But this may also suggest that these rats felt safe, because they overcame the challenge of finding new routes through the maze each time its configuration was changed.

Rats with SRS raised in Marlau™ cages exhibited reduced anxiety compared to their counterparts raised in conventional cages. However, increase in anxiety was the same between the two housing conditions when rats with SRS were compared with their respective controls. This result may correspond to the development of an adaptive behavior in all rats with SRS, since seizures, with loss of consciousness, may represent a great source of danger. Indeed, anxiety has been proposed as a protective mechanism preventing the organism from engaging into potentially harmful behaviors [100], [101].

In conclusion, the Marlau™ cage, by providing standardized enrichment procedures for rodents during housing, should facilitate transfer of reproducible programs of environmental enrichment across laboratories. This implies that the changes in maze configuration and in bedding material must strictly occur across studies as described. Standardization of enrichment procedures appears now to be crucial, since interest in gene/environment interactions extends far beyond the field of neuroscience research, including immunology and cancer research for instance [5], [6], [7].

## Materials and Methods

### Ethics Statement

All animal procedures were approved by the Animal Care and Use Committee of University Claude Bernard Lyon 1, and were in compliance with the guidelines of the European Union (directive 86/609 and revisited Appendix A of the ETS123), taken in the French law (decree 87/848) regulating animal experimentation. Throughout all experiments using either naïve control rats or rats subjected to *status epilepticus*, all efforts were made to reduce the number of animals used. We performed a daily (except on Sundays) observation of all animals and measured the body weight twice a week to detect any change in the general state of the animals. In rats subjected to *status epilepticus,* duration of continuous behavioral seizures was maintained as short as possible (35 min) to induce delayed epileptic seizures in the majority of the animals. During *status epilepticus,* about 5–10% of the rats died after a respiratory arrest related to a tonic seizure. After *status epilepticus*, body weight was measured daily until rats gained weight. If weight gain did not occur within four days following the initiation of *status epilepticus*, rats were sacrificed. Thereafter, all rats were daily observed as stated above. No rat subjected to *status epilepticus* suddenly or unexpectedly died during and after the recovery period. Nevertheless, humane end points had been anticipated and defined as follows: 1) any decrease in body weight exceeding 10%; 2) any development of aggressive behavior or auto-mutilation. At termination time, all rats were sacrificed after a lethal injection of pentobarbital (250 mg/kg). Finally, regarding the minimization of animal suffering, a main objective to develop the Marlau™ cage was to increase animal’s wellbeing throughout experimentations [13].

### Animals

Male Sprague-Dawley rats (Harlan, France) were used in these experiments. Pups arrived at 15 day-old with their foster dams, and were maintained in groups of 10 in plastic cages with free access to food and water. At 21 days of age, rats were weaned and housed either in conventional (Cv) cages (type “E”, length 405 mm, depth 255 mm, height 197 mm, total exploration surface = 800 cm^2^; Charles River, France) or in the enriched Marlau™ cage (En) for up to 13 weeks. Animals were housed in groups of 6 in conventional cages and in groups of 12 in Marlau™ cages at 21°C under diurnal lighting conditions (lights on from 06∶00 to 18∶00). In both types of cage, aspen wood bedding material was changed once per week on Fridays at 16∶00. The aspen wood bedding was half Litaspen Premium 6 and half Litaspen Premium 8/20. All rats were fed with the same food pellets (type A04, Safe). All animals were weighed twice each week, and removed from their cage when bedding material was changed.

### Marlau™ Cage

This cage was engineered and patented (FR2941844) by our laboratory to standardize procedures of enrichment for rodents, as already described in great details [13].

### Experimental Design of Animal Studies

#### Experiment 1

Overall physiological status: body weight, food intake and stress-coping. In this experiment, 12 rats were housed in one Marlau™ cage and 12 rats were housed in two conventional cages (6 rats per cage) for a period of six weeks. All rats were weighed twice per week, and food pellets were weighed once a week in each cage at the time of bedding change. In order to investigate the impact of housing conditions on stress coping ability, plasma CORT concentrations were measured under basal conditions and in response to restraint stress in 10 randomly chosen rats (with 5 in each Cv and En groups). The 7 remaining rats in each group were used to assess hippocampal GR and MR receptor transcript levels, and total lipids in the whole body (without brain tissue).

#### Experiment 2

Histological and biochemical characterization of the enrichment procedures using the Marlau™ cage. Four weeks after weaning, 4 of the “En” rats, and 2 “Cv” rats in each of the 2 conventional cages were injected with BrdU, as described below. The other rats received vehicle. Two weeks later, rats injected with BrdU (8 in total) and controls were prepared for histological analysis (hippocampal neurogenesis, cortical thickness). The other rats (n = 8 in each Cv and En groups) were used to assess gene expression in the hippocampus (Hi) at transcript level using RT-real time PCR (RT-qPCR).

#### Experiment 3

Testing the repeatability of the results obtained in Marlau™ cage. The experiment has been repeated twice: Exp.3a and Exp.3b, with 12 rats of each Cv and En conditions in each experiment (n = 48 in total). Rats were subjected at 6 weeks to the Morris Water Maze test (MWM), and then sacrificed two weeks later to assess gene expression in the ventral hippocampus at transcript level using RT-qPCR.

#### Experiment 4

Longitudinal analysis of exploratory behavior. After weaning, rats (n = 24, with 12 in each Cv and En groups) were subjected to three different tests, as described below, performed at a different developmental ages: WET, BWB box test, and finally EPM test at 4, 6 and 15 week-old, respectively.

#### Experiment 5

Validating WET as a way to measure anxiety-like behavior. After weaning, 36 rats were housed in groups of 6 in 6 type “E” cages. Six weeks later, rats were transferred into individual cages 3 hours prior to i.p. administration of either 25 mg/kg FG-7142 (β-carboline-3-carboxylic acid N-methylamide; Sigma) (n = 12) or 3 mg/kg diazepam (n = 12), already shown to produce anxiogenic or anxiolytic effects in rats at these doses [54], [55], respectively. Control rats were injected with saline (control saline, n = 6) or with dimethyl-sulfoxide (DMSO) (control DMS0, n = 6). Because no difference was observed between the two control groups, they were pooled together and called as “controls”. Rats were tested in the WET 30 min later. In addition, since housing in Marlau™ cages reduces anxiety-like behavior (experiment 4), we tested in rats housed for 6 weeks in Marlau™ cages after weaning whether anxiogenic effect of FG-7142, administered as above, was easier to demonstrate. Twenty-four rats were raised in 2 different Marlau™ cages, and six rats of each cage was randomly assigned to either FG-7142 or DMSO administration.

#### Experiment 6

Testing the effect of Marlau™ cage on LTP in the hippocampus in healthy rats and in rats subjected to lithium-pilocarpine-induced status epilepticus (Li-Pilo-SE). At weaning, rats (n = 50) were subjected to Pilo-SE (n = 26) and were housed, 1 day later, in two conventional cages (n = 6 in each cage) and one Marlau™ cage (n = 12). Two rats died during Pilo-SE. Control rats (n = 24) were housed in two additional conventional cages (n = 6 in each cage) and in one other Marlau™ cage (n = 12). Cellular mechanisms underlying memory in the hippocampus (LTP) were monitored 1–2 weeks post-SE.

#### Experiment 7

Testing the protective effect of Marlau™ cage against cognitive impairment in epileptic rats. At weaning, rats (n = 60) were subjected to Li-Pilo-SE (n = 40) and were housed 1 day later, in 5 conventional cages (total n = 18 with n = 3−4 in each cage) and two Marlau™ cages (total n = 18 with n = 9 in each cage). Four rats died during Pilo-SE. Control rats (n = 20) were housed in two additional conventional cages (total n = 10, n = 5 in each cage) and in one other Marlau™ cage (n = 10). Cognitive performance was assessed at 15 and 16 weeks using the WET and the MWM, respectively, in rats that developed spontaneous recurrent seizures (n = 16 in conventional cages and n = 9 in Marlau™ cages) and in controls.

### *In Vivo* Procedures

#### BrdU administration

At 7 weeks of age (4 weeks after weaning), rats received 5 consecutive injections of 5′-bromodeoxyuridine (BrdU; Sigma B5002, 50 mg/kg, i.p.), at 17∶00 the first day, and at 12∶00 and 17∶00 on the two following days, as previously described [24]. Rats were sacrificed two weeks later.

#### Acute restraint stress protocol

After 6 weeks of housing conditions, rats (n = 5 in each Cv and En groups) were placed in well-ventilated plexiglass restraint tubes for 30 min. At each blood sample collection, animals were anesthetized with halothane and blood (250–300 µL) was collected retro-orbitally in EDTA tubes (BD Microtainer K_2_E) to determine CORT level. Samples were collected at basal level, at the end of restraint stress, and after 2 hours of recovery. Blood samples were centrifuged for 5 min at 10,000 rpm, and plasma was kept at −20°C.

#### Morris Water Maze (MWM)

A circular tank (180 cm diameter, 60 cm high) was filled with water maintained at 25°C to a depth of 40 cm. The water was made opaque by addition of black gouache, which prevented visualization of the platform by the rat. All visual clues around the room were kept in a constant location from day to day. The pool was divided into 4 virtual quadrants defined as North (N), East (E), South (S), and West (W). A circular platform (10 cm diameter) was hidden 1 cm below the surface of the water, and was kept at a constant position within the northern quadrant, close to the NW border. Rats were tested for a total of 16 trials performed during 4 consecutive days. Each day, rats were subjected to 4 trials, at 2-hour intervals, each trial lasting 90 sec. Rats were videotracked by a camera positioned on the ceiling just above the center of the tank, using software (Videotrack, Viewpoint) that calculated the distance and the time to find the platform. Animals were introduced into the water facing the wall of the pool at a position that was changed at each trial as follows: 1^rst^ day, ESWE; 2^nd^ day, SWES; 3^rd^ day, WESW; 4^th^ day, ESWE. When rats found the platform, they were allowed to stay on it for 15 sec. When they did not find the platform, they were placed on it for 15 sec. Investigators were not in the room during the tests.

#### Water Exploration Test (WET)

The tank used for the MWM was divided into four identical quadrants (6,358 cm^2^ each) using two 180 cm long opaque plastic separators. Four rats were tested for 5 min at the same time, but in different quadrants. The VideoTrack system calculated the time, the distance, and the velocity of exploration within the full quadrant, and within a virtual central zone of 2,921 cm^2^ (Fig. 7.A.a).

#### Black/White/Black box test (BWB)

This test was inspired by the Dark/Light box test [102]. The box (Fig. 7.B.a) consisted of two black compartments of different sizes, 800 cm^2^ and 600 cm^2^, separated by a larger white compartment (1,000 cm^2^). Rats were always introduced into the same corner of the large black compartment. Animals had free-access to the white compartment. A gate, easily removed by the investigator, prevented access to the small black compartment from the white compartment. On the first day of the test, rats had only access to the large black and white compartments. On the second day of the test, rats had access to all three compartments. Test duration was 90 sec on both days. The VideoTrack system calculated the time spent and the distance traveled in each compartment.

#### Elevated Plus Maze test (EPM)

The maze (Fig. 7.C.a) consisted of two open arms (OA) (50×15 cm) perpendicularly positioned to two arms (50×15 cm) closed by 40-cm high walls. The maze was elevated 60 cm above the floor. Rats were isolated 30 min in a conventional cage (type “E”) before the test. Each animal was placed for 5 min in the central platform (15×15 cm), facing the same OA. The maze was cleaned with tap water and dried after each trial to eliminate possible odor cues left by previous rats. All rats were video monitored and the following variables were measured: the number of entries, the time spent and the distance traveled in each arm. Entries were counted whenever rats positioned their four paws into a new arm.

#### Pilocarpine-induced status epilepticus

On day 21, pups were injected with lithium chloride (Sigma, 3 mEq/kg, i.p.) freshly dissolved in saline. Eighteen hours later, rats were given scopolamine methylnitrate (Sigma, 1 mg/kg s.c.) followed, 30 min later, by pilocarpine hydrochloride (Sigma, 25 mg/kg, i.p.). After 35 min of continuous behavioral SE, a single injection of diazepam (Valium®, 10 mg/kg, i.p; Roche) was administered to terminate behavioral seizures. Controls received 4 saline injections.

#### Spontaneous seizure detection in rats subjected to status epilepticus

Electrode implantation for continuous EEG monitoring was not possible in our social housing conditions, in particular when using the Marlau™ cages. Thus, to identify rats that indeed developed spontaneous recurrent seizures from the 10^th^ to the 13^th^ week after SE, rats were observed by two persons, 8 hrs a day (from 08∶00–12∶00 and from 13∶00–17∶00), 5 days a week (from Mondays to Fridays).

### *Ex Vivo* Procedures

All rats were deeply anesthetized with a lethal dose of pentobarbital (250 mg/kg) before being sacrificed. For biochemical analysis, brain structures were rapidly microdissected, frozen in liquid nitrogen and stored at −80°C. For immunohistochemistry analysis, animals were transcardially perfused (30 mL/min) with chilled 4% paraformaldehyde in 0.1 M phosphate buffer. After cryoprotection in 25% sucrose, brains were frozen at −40°C in isopentane and stored at −80°C.

#### Total body fat determination

After brain removal, the rest of the body was weighed and digested in hot 30% KOH. Aliquots (5 g) of the saponified samples were neutralized with concentrated HCl; lipids were extracted and washed as previously described [103]. Briefly, the lipids were extracted overnight at 4°C in chloroform-methanol (2∶1, v/v) and washed twice by addition of 0.25% (w/v) aqueous KCl solution. The chloroform lower phase was evaporated to dryness until the mass of lipids remained constant. Total lipid content was expressed as the percentage of lipid mass/rat body weight.

#### Quantitative determination of CORT using Elisa

CORT was extracted from plasma samples and measured using an Elisa kit (Neogen Corporation) following manufacturer’s instruction.

#### Quantitation of transcript level variations by RT-qPCR

Variations in transcript levels were determined by real time PCR amplification of cDNAs of interest after reverse transcription (RT) of total mRNAs, as previously detailed [24], [104], [105], [106]. A synthetic external and non-homologous poly(A) Standard RNA (SmRNA) was used to normalize the reverse transcription of mRNAs of biological samples (Morales and Bezin, patent WO2004.092414). Sequences of the different primer pairs used are: ***BDNF*** (GenBank accession no. X67108) forward 5′ AAA TTA CCT GGA TGC CGC AA 3′, reverse 5′ CGC CAG CCA ATT CTC TTT TT 3′ (345 bp); ***Epo*** (GenBank accession no. NM_017001) forward 5′ GCT CCA ATC TTT GTG GCA TC 3′, reverse 5′ ATC CAT GTC TTG CCC CCT A 3′ (66 bp); ***EpoR*** (GenBank accession no. D13566) forward 5′ CCA GCT CTA AGC TCC TGT GC 3′, reverse 5′ CTT CAG GTG AGG TGG AGT GG 3′ (68 bp); ***GR*** (GenBank accession no. AY293740) forward 5′ CAC AAG CAA TGT GCA G 3′, reverse 5′ AAG TGA AAC GGC TTT GGA TAA G 3′ (103 bp); ***IGF-1*** (GenBank accession no. NM_178866.2) forward 5′ATG CCC AAG ACT CAG AAG GA 3′, reverse 5′ CGT GGC ATT TTC TGT TCC TC 3′ (110 bp); ***MAP-2*** (GenBank accession no. NM_013066) forward 5′ GTG TTA AGC GGA AAA CCA CAG 3′, reverse 5′ GAC TTT GTC CTT CGC CTG TT 3′ (80 bp); ***MR*** (GenBank accession no. M36074.1) forward 5′ TGA AGG TTT TGC TGC TAC TAA GC 3′, reverse 5′ TGT AAT TTG TCC TCA TCT CCT CAA 3′ (84 bp); ***TrkB.FL*** (GenBank accession no.M55291) forward 5′ TGA AGA CGC TGA AGG ACG CCA 3′, reverse 5′ CAG GTT CTC TCC TAC CAA GCA 3′ (353 bp); ***TrkB.T1*** (GenBank accession no. M55292) forward 5′ CTG GAT GGC TAG CTG AGA TAA AGG A 3′, reverse 5′ AGT CAC AGC TCA CAA CAA GCA GGC T 3′ (187 bp); ***TrkB.T2*** (GenBank accession no. M55293) forward 5′ TAC TCA GCC TTG CCC ACT TT 3′, reverse 5′ GCC ATA ACA TAT CCT TGC CC 3′ (162 bp); ***VEGF*** (GenBank accession no. NM_031836) forward 5′ CCT GTG TGC CCC TAA TGC 3′, reverse 5′ AGG TTT GAT CCG CAT GAT CT 3′ (107 bp).

#### Fluorescent detection of BrdU-positive cells in the hippocampus

Free-floating coronal cryostat sections (40 µm thick) were mounted on SuperFrost®Plus slides and air dried. After DNA denaturation for 30 min in 2M HCl at 65°C and neutralization in borate buffer pH 8.5, sections were incubated overnight at 4°C with a rat monoclonal antibody raised against BrdU (OBT-0030; Oxford Biotechnology) diluted at 1/25, and then with an Alexa-488-conjugated donkey anti-rat IgG antibody (A21208; Molecular Probes) diluted at 1/1000. In dual immunolabelings, astroglial marker GFAP or neuronal marker NeuN was detected together with BrdU, using mouse monoclonal antibodies raised against GFAP (G3893, Sigma) and NeuN (MAB377, Chemicon) diluted at 1/1000, respectively. In this case, anti-BrdU antibody was detected as above, and mouse monoclonal antibodies raised against GFAP or NeuN were recognized using an Alexa-633-conjugated goat anti-mouse IgG antibody (A21052; Molecular Probes) diluted at 1/2000. Sections were then analyzed under the same conditions of photomultiplier gain, offset, and pinhole aperture using a TCS SP2 confocal microscopy system (Leica), allowing the comparison of fluorescence intensity between regions of interest. Images were imported into Adobe Photoshop 8.0.1 (Adobe Systems).

### Electrophysiology

#### Slice preparation

Transverse hippocampal slices were prepared from postnatal day 28–38 Sprague-Dawley rats. Animals were anesthetized and killed by decapitation and the brain is quickly removed and cooled with ice-cold standard artificial cerebrospinal fluid (ACSF) containing the following (in mM): 124 NaCl, 5 KCl, 1.25 Na_2_HPO_4_, 2 MgSO_4_, 2 CaCl_2_, 26 NaHCO_3_, and 10 D-glucose, bubbled with 95% O_2_ and 5% CO_2_. Hippocampi were dissected out, and transverse slices (400 μm thickness) were cut on a vibratome (Leica VT1000S) equipped with a ceramic blade. The slices were then transferred to ACSF at room temperature for at least 1 hr before transfer to the recording chamber. The ACSF used for perfusion is supplemented with 100 μM picrotoxin to block GABA_A_ receptors.

#### Electrophysiological recordings

Whole-cell patch-clamp recordings were obtained from CA1 pyramidal neurons in current clamp mode at −70 mV with a patch pipette (3–5 MΩ) containing (in mM): 120 potassium gluconate, 20 KCl, 0,2 EGTA, 2 MgCl_2_, 10 HEPES, 4 Na2ATP, 0.3 Tris-GTP and 14 mM phosphocreatine (pH 7.3, adjusted with KOH). All drugs were purchased from Sigma. Hippocampal CA1 pyramidal neurons were visualized with a Zeiss Axioskop 2 equipped with 40 objective, using infrared video microscopy and differential interference contrast optics. Series resistance (typically 15–25 MΩ) was monitored throughout each experiment; cells with more than 15% change in series resistance were excluded from analysis.

Whole-cell patch-clamp recordings were performed using an Axopatch-200B amplifier (Molecular Devices) at the sampling rate of 10 kHz and filtered at 5 kHz. Data were acquired and analyzed using a Digidata 1440A interface and pClamp 10 software (Molecular Devices). Capillary glass pipettes filled with ACSF and connected to an Iso-Flex stimulus isolation unit (A.M.P.I.) were used to stimulate presynaptic axons in stratum radiatum (120–150 mm away from the soma). Stimulation at 0.05 Hz was used to establish baseline synaptic responses. The stimulation strength was set to evoke EPSPs between 5–8 mV.

LTP was induced using the theta burst pairing (TBP) protocol, which was delivered within 20 min after whole-cell formation to prevent the washout of LTP induction. Back propagating action potentials (b-APs) were elicited by direct somatic current injection (1 ms, 1–2 nA). The standard TBP protocol consists of EPSPs paired with a single b-AP timed so that the b-AP (∼15 ms delay) occurred at the peak of the EPSPs as measured in the soma. A single burst contained five pairs delivered at 100 Hz and ten bursts were delivered at 5 Hz per sweep. Three sweeps were delivered at 10 s intervals for a total of 30 bursts (150 backpropagating action potential-EPSP pairs). Electrophysiological data were analysed using pClamp 10 and Igor pro software (WaveMetrics).

### Morphological Analysis

#### Measurement of cortical thickness and hippocampus volume

After sections were Nissl-stained, images were captured with a video camera 3CCD (DXC-9300; Sony) coupled to an image analysis system (Visilog 6.3; Noesis). Cortical thickness was measured as shown (Fig. 3A) at IA 9.70 mm and 5.40 mm [18]. The surface area of both sides of the hippocampus (dorsal anterior, daHi; dorso-ventral, dvHi) was measured from IA 5.86 mm to 4.70 mm. Results are expressed as the mean ± SEM of the surface areas obtained from 4 sections (daHi) and 2 sections (dvHi).

#### BrdU-positive cell counting

For each brain, we analyzed three sections containing the dorsal hippocampus, selected at IA 5.70 mm, 5.40 mm and 5.10 mm [18]. The granule cell area was traced using an image analysis system (Visilog 6.3; Noesis). The number of BrdU-labeled cells was counted in this delimited area for all sections by an investigator blind to the group (Cv and En).

### Statistical Analysis

Data are expressed as mean ± SEM of the different variables analyzed. For body weight data, we calculated individual growth taking the body mass at the age of 28 days as reference point. For each rat, the value of each of the weightings has been expressed as a percentage of the value measured at the age of 28 days. Since a significant interaction has been found between “housing conditions” (Cv and En) and “age” using two-way repeated measures ANOVA, body weight gain was plotted against age and linear regression were established for each group of rats (Cv and En). To determine whether the slope difference was significant, we established for each rat a linear regression between body weight gain and age and tested slope difference between the two groups of rats with a Student’s *t*-test. Differences in plasma corticosterone level after restraint stress, in performances in the Morris Water Maze and in amplitude of LTP were tested using two-way repeated measures ANOVA 2. Differences in cortical thickness, in transcript level, in anxiety-like behavior in the WET in epileptic rats were tested using a two-way ANOVA. Finally, differences in anxiety-like behavior in the WET and in the elevated plus maze in naïve rats housed in Cv or in En cages were tested using a Student’s *t*-test. Two-way ANOVA were followed by *post-hoc* Fisher’s protected Least Significance Differences (LSD) test or the Mann–Whitney *U* test (LTP experiments exclusively).
